# Study of Bitespiramycin Distribution in Rats and Cerebrospinal Fluid of Patients by a Sensitive LC-MS/MS Method with Rapid Sample Preparation

**DOI:** 10.3390/molecules29051037

**Published:** 2024-02-28

**Authors:** Yujie Zhang, Jingjie Cao, Jiahan Su, Tingting He, Qianru Wang, Feng Wei, Xin Guo, Qibing Mei, Jing Zeng

**Affiliations:** 1 School of Pharmacy, Southwest Medical University, Luzhou 646000, China; yujiezhang_995@163.com (Y.Z.);; 2 Shanghai Tonglian Pharmaceutical Co., Ltd., Shanghai 201611, China; 3 Luzhou New Drug Safety Evaluation Research Center, Luzhou 646000, China

**Keywords:** bitespiramycin, distribution, LC–MS/MS, rat, human cerebrospinal fluid

## Abstract

Bitespiramycin, has been shown to have a therapeutic effect against respiratory tract inflammation, including a potential effect against COVID-19. A current clinical trial in China showed that bitespiramycin was an effective treatment for severe pneumonia and intracranial infection. However, there is lack of an analytical method to elucidate the distribution of bitespiramycin. In this study, a highly sensitive, rapid and reliable UPLC–MS/MS method was developed to comprehensively characterize the bitespiramycin distribution in various bio-samples, which is significantly improved upon the published work. A rapid sample preparation method was developed by using *n*-butanol as the solvent to extract bitespiramycin from different bio-samples. The extract was then directly analyzed by UPLC–MS/MS coupled with an alkaline-resistant column after centrifugation which avoids the time-consuming concentration process under nitrogen and redissolution. The method was employed to accurately quantify bitespiramycin and its metabolites in rat plasma, tissues, and human cerebrospinal fluid. Notably, the presence of bitespiramycin and its metabolites was identified for the first time in various rat organs including brain, testis, bladder and prostate as well as in human cerebrospinal fluid. This newly developed approach shows great promise for drug distribution assays including other antibiotics and can help elucidate the ADME of bitespiramycin.

## 1. Introduction

**Figure 1:**
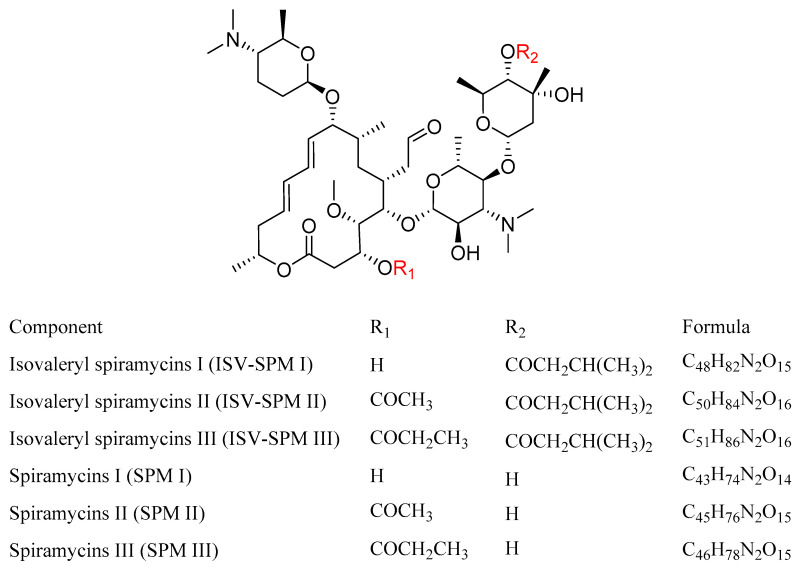
The structures and formulas of bitespiramycin and its metabolites.

Bitespiramycin, a new 16-membered macrolide antibiotic, has been shown to have excellent therapeutic effect against respiratory tract inflammation, including the potential effect against COVID-19 [1]. It has been listed in a clinical trial (ClinicalTrials.gov Identifier: NCT04672564) to validate the efficacy against COVID-19 by National Institutes of Health (NIH) [2]. In addition, isovaleryl spiramycins I (ISV-SPM I), the main component of bitespiramycin, has been proved to be an active ingredient to suppress cancer cell growth and tumor metastases by targeting selenoprotein H [3]. Different from other drugs with a single ingredient, bitespiramycin is mainly composed of ISV-SPMs I, II, and III [4], which could interact with multiple targets in microenvironments. The three pharmacologically active ingredients can rapidly undergo in vivo de-esterification, resulting in the formation of spiramycins (SPMs) I, II, and III as metabolites (Figure 1) [5]. These three metabolites exhibit a strong positive correlation with therapeutic efficacy.

Recently, a new clinical trial in China has shown that bitespiramycin has excellent therapeutic effect against severe pneumonia, prostatitis, and intracranial infection (unpublished data). However, comprehensive distribution of bitespiramycin in tissues and body fluids, especially in lung, prostate, and cerebrospinal fluid needs to be further elucidated. An applicable analytical method with simplicity, high sensitivity and specificity is urgently needed, since the lowest concentration of bitespiramycin in some tissues would be in the range of ppb to ppt.

LC–MS/MS-based quantitation methods were usually used to determine metabolic profiles in vivo, tissue distribution, and pharmacokinetics of bitespiramycin in recent years, while sample preparation was very time-consuming [6,7,8,9]. Reversed phase (C_18_)—based solid phase extraction (SPE) combined with being concentrated under a stream of nitrogen and redissolution was used to prepare body fluids, including plasma, urea, and bile. In addition, a mixed solvent, ethyl acetate–isopropanol (95:5, *v*/*v*) based liquid–liquid extraction (LLE) method at pH 9~10 was developed to extract bitespiramycin and its metabolites in tissues, followed by being concentrated and redissolved before LC–MS/MS measurement. In another study, acetonitrile and ethyl acetate were employed as the protein precipitating and extracting agent respectively in the study of bitespiramycin pharmacokinetics in beagle dogs [10]. The extract was then concentrated and redissolved before UHPLC–MS/MS analysis, which was similar to the previous studies [6,7].

In this study, a rapid, sensitive and reliable UHPLC–MS/MS method, specifically to simplify the sample preparation, detect bitespiramycin and its metabolites simultaneously in rat plasma, tissues, and human cerebrospinal fluid, was developed. It revealed the underlying mechanism of the clinical therapeutic effect on severe pneumonia, prostatitis, and intracranial infection. The newly developed approach could provide a promising strategy to analyze macrolide antibiotics in vivo.

## 2. Results and Discussion

### 2.1. Method Development

Sample extraction, purification, and enrichment are often the rate-limiting steps of an analytical method, making them critical aspects of method development. As for the extraction solvent selection, acetonitrile, ethyl acetate, chloroform, dichloromethane, and *n*-butanol are commonly used solvents for sample extraction. Acetonitrile is a well-known solvent for protein precipitation, but it is not functional for the isolation of liposoluble compounds (phospholipids, etc.), resulting in rapid decrease of efficiency and increase of back pressure of columns. Chloroform and dichloromethane are good choices for the LLE, but not compatible with the aqueous mobile phase in the UHPLC separation. Another option for sample extraction is a mixed solvent composed of acetonitrile and ethyl acetate, followed by several steps including vortexing, centrifugation, nitrogen concentration, and redissolution [9,10]. N-butanol is a popular solvent to extract polar or medium-polar components in the area of phytochemistry, which would be a potential proper solvent for the extraction of bitespiramycin and its metabolites. In this study, the solubility of bitespiramycin in *n*-butanol was found to be about 2 times higher than that in ethyl acetate. The boiling point of *n*-butanol (117 °C) is much higher than other solvents and less volatile, resulting in the concentration of *n*-butanol extract being more stable. Moreover, a microliter of *n*-butanol extract is soluble in the aqueous mobile phase, facilitating direct analysis of the extract. Therefore, *n*-butanol was selected as the extraction solvent in this study. Bio-samples extracted by *n*-butanol could be directly injected to the UHPLC–MS/MS system without being further concentrated under nitrogen and redissolution, which reduced the whole process time and significantly improved the analytical throughput and stability.

The pH of the mobile phase is widely recognized to have a significant impact on separation and detection processes. Bitespiramycin, being a weak alkaline compound due to its amino sugar moiety, typically exhibits low efficiency or peak tailing when subjected to acidic mobile phase conditions owing to the strong ion interaction between dissociated silanol (anions) and bitespiramycin (cations) [7,8,9,10]. Under the condition of alkaline mobile phase (pH_mobile phase_ > pI_bitespiramycin_), bitespiramycin is a kind of neutral molecules, and the strong ion interactions were greatly reduced, improving peak shape and column efficiency. Therefore, the alkaline mobile phase (pH 8.8) coupled with Waters Acquity BEH–C_18_ column with the widest pH range (pH 1–12) was used. The peak shapes of bitespiramycin and internal standard azithromycin were significantly improved, which increases the sensitivity of this method. Additionally, UHPLC gradient, parameters of ion source, MRM transitions and collision energy were carefully optimized. As a result, a sensitive LC-MS/MS method with rapid sample preparation was successfully developed.

### 2.2. Method Validation

#### 2.2.1. Specificity and Selectivity

**Figure 2:**
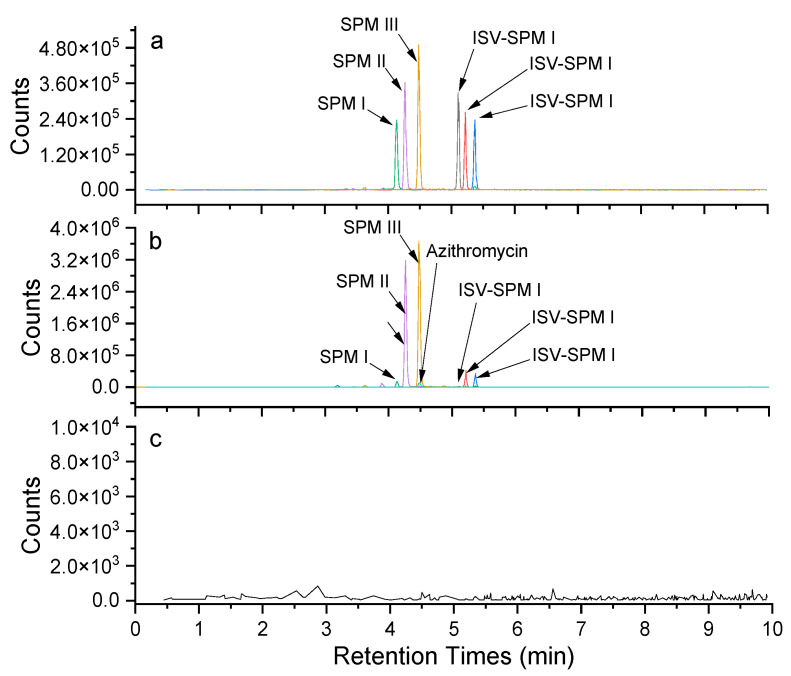
The MRM chromatograms of mixtures of six standards (**a**), plasma after bitespiramycin administration and spiked internal standard azithromycin (**b**), and blank plasma (**c**).

The MRM chromatograms of a mixture containing six standards, plasma samples after administration of bitespiramycin and spiked internal standard azithromycin were overlaid, as depicted in Figure 2a and Figure 2b, respectively. Each MRM chromatogram exhibited a single peak in its molecular ion−product ion transition without interference from endogenous impurities due to the high resolution and specificity of the triple quadrupole mass analyzer. However, it should be noted that the six components exhibited baseline separation with a minimum resolution of approximately 1.5. Furthermore, Figure 2c displays the MRM chromatograms of blank plasma without any interfering peaks observed, indicating exceptional specificity and selectivity.

#### 2.2.2. Linearity and LLOQ

**Figure 3:**
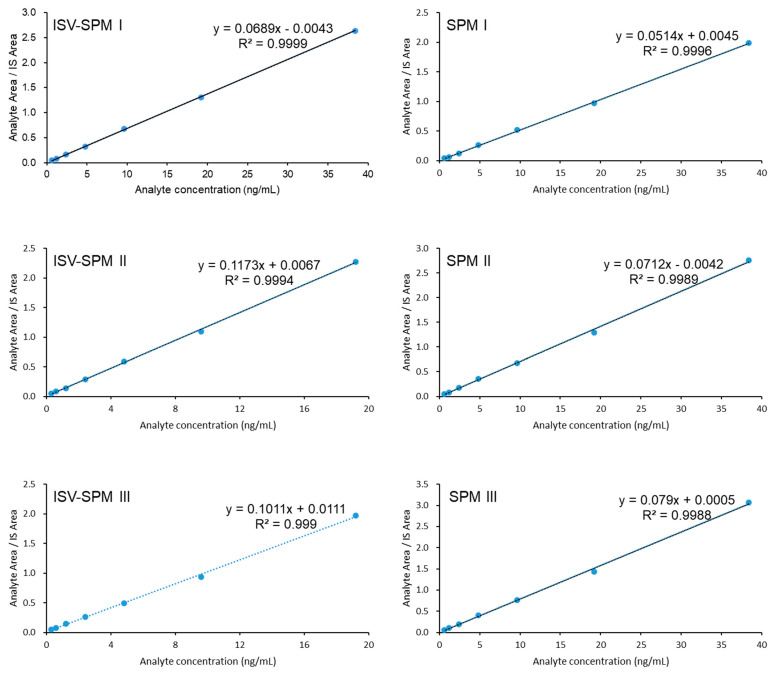
The Linearity of SPMs I, II, III, and ISV-SPM I, II, III in cerebrospinal fluid of patients.

Six standards spiked in rat plasma, tissues and cerebrospinal fluid of patients with different concentrations were measured, and the representative linear curve of cerebrospinal fluid was plotted in Figure 3. The linear ranges were shown in Table 1. The concentration (X) is linearly related to the ratio of analyte to internal standard (Y). The deviation between the actual concentrations with standards at LLOQ (signal to noise = 10) was within 20%, and the deviations of other concentration were all within ±15%. The LLOQ was significantly improved in this study compared to the LLOQ published in the literature [9,10], as shown in Table 2.

#### 2.2.3. Accuracy and Precision

The accuracy fell within the range of 89.90% to 111.10%. The intra-day and inter-day precision errors were below 13.62% and 13.71%, respectively, indicating compliance with the specifications outlined in the Chinese Pharmacopoeia (Guidelines for Validation of Quantitative Analysis Methods for Biological Samples).

#### 2.2.4. Extraction Recovery Rate and Matrix Effect

The extraction recovery rate of bitespiramycin and internal standard varied from 85% to 115%. The ion suppression/enhancement was less than 15% for QC samples, indicating good accuracy and reproducibility.

#### 2.2.5. Stability

RSD of ISV-SPMs I, II, III and SPMs I, II, III under four storage conditions were all less than ±15%, indicating bitespiramycin and its metabolites were stable.

### 2.3. Distribution of Bitespiramycin in Rats

#### 2.3.1. Distribution in Rat Plasma

**Figure 4:**
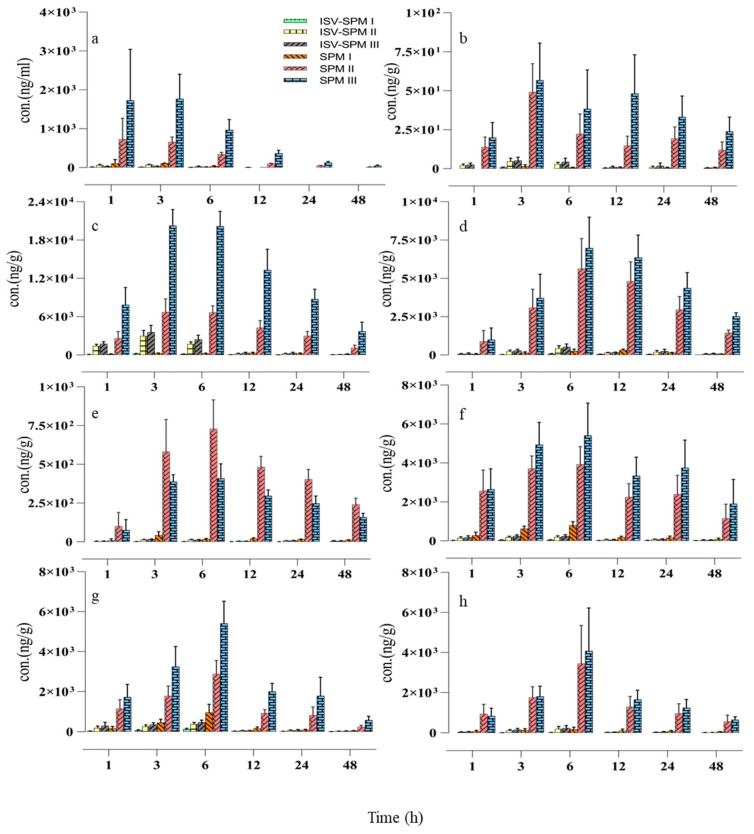
The distribution of SPMs I, II, III and ISV-SPMs I, II, III in the plasma (**a**), brain (**b**), lung (**c**), prostate (**d**), testis (**e**), womb (**f**), ovary (**g**), and bladder (**h**) of rats after intragastric administration of 100 mg/kg of bitespiramycin (Mean ± SD, *n* = 6).

Using the validated UHPLC–MS/MS method, bitespiramycin and its metabolites concentration in rat plasma was measured at 1, 3, 6, 12, 24, and 48 h post intragastric administration of 100 mg/kg of bitespiramycin. As shown in Figure 4a and Table S1, ISV-SPMs I, II, and III were rapidly metabolized to SPMs I, II, and III, respectively. The concentration of ISV-SPMs I (5.08 ng/mL), II (25.05 ng/mL), and III (13.15 ng/mL) after 6 h administration remained at lower levels, while that of SPMs I (33.65 ng/mL), II (338.67 ng/mL), and III (970.00 ng/mL) remained at significantly higher levels, indicating that the three metabolites of bitespiramycin would be the pharmacologically active metabolites. After 48 h post-administration, the ISV-SPM I in plasma cannot be detected, while the concentration of SPM III remained at 47.12 ng/mL.

#### 2.3.2. Distribution in Rat Tissues

The concentrations of six analytes in tissues were also measured at 1, 3, 6, 12, 24, and 48 h after intragastric administration of 100 mg/kg of bitespiramycin, shown in Figure 4b–h and Tables S2–S7. ISV-SPMs I, II, III and SPMs I, II, III were absorbed, distributed, accumulated and eliminated in various tissues, including lung, uterus, ovary, testis, prostate, bladder and brain. Their concentrations in these tissues were significantly different. Concentrations of ISV-SPMs I, II, III and SPMs I, II, III in lung were the highest, which was about 200 times more than that in the lowest tissue, the brain. The highest concentration of ISV-SPMs I, II, III and SPMs I, II, III in these tissues was observed at 6 h, except in the brain which was at 3 h.

These results indicated that ISV-SPMs I, II, III and SPMs I, II, III tend to distribute in lung, followed by prostate, bladder, womb, ovary, testis, brain and cerebrospinal fluid. Bladder, prostate and cerebrospinal fluid of patients were examined for the first time. It was also the first time that bitespiramycin was successfully detected in brain and testis due to the significantly improved sensitivity of this method. Furthermore, after comparing the concentrations of SPMs and ISV-SPMs, it can be concluded that the concentrations of SPMs in tissues were about 10 times higher than that of ISV-SPMs, indicating that parent drugs, ISV-SPMs I, II, and III were rapidly metabolized to SPMs I, II, III, respectively, similar to the results in plasma. Moreover, SPMs I, II, III were eliminated slowly, which cannot completely be cleared from the rat’s body, even after 48 h. It was interesting that the concentration of SPM III in lungs was as high as 3700.33 ng/g after 48 h, which would directly contribute to the therapeutic efficacy against pneumonia. The highest concentration of ISV-SPMs I, II, III and SPMs I, II, III in these tissues was observed at 6 h, except in the brain which was at 3 h.

The tissue-to-plasma concentration ratio (*C_t_*/*C_p_*) at 1, 3, 6, 12, 24, and 48 h was statistically analyzed in Table 3 and Tables S9–S13. At the 24 h post-dose time point, ISV-SPMs II, III and SPMs I, II, III were detected except for ISV-SPM I. There were significant differences in the concentrations of six components in various tissues. The average *C_t_*/*C_p_* value in the brain was found to be 0.51, whereas it was significantly higher in the lung (104.86), followed by the prostate (82.06), womb (46.55), ovary (30.11), bladder (22.11), and testis (4.88). These findings indicate that bitespiramycin exhibits extensive tissue distribution with the exception of in the brain.

### 2.4. Distribution in Cerebrospinal Fluid of Patients

To further identify if bitespiramycin could cross the blood-brain barrier, bitespiramycin and its metabolites in cerebrospinal fluid of patients were measured. As shown in Table 4, the concentration of the six analytes collected on the first day was in the range of 0–1.36 ng/mL and on the fifth day in the range of 0–3.40 ng/mL, respectively, indicating a trend of drug accumulation. Moreover, the concentrations of the six analytes in another sample collected on nineteenth and twentieth days increased to the range of 0–13.00 ng/mL, which was about 5 times higher than that on the fifth day. Bitespiramycin and its metabolites were definitively accumulated in the brain to a relatively high concentration, due to the properties of slow elimination.

## 3. Material and Methods

### 3.1. Reagents

Bitespiramycin Tablets (Shanghai Tonglian Pharmaceutical Co., Ltd., Shanghai, China, Lot No. 0220200204); SPMs I, II, III and ISV-SPMs I, II, III standards (Shanghai Tonglian Pharmaceutical Co., Ltd., purity 98%); Acetonitrile (chromatographic grade; Fisher Chemical, Waltham, MA, USA); *n*-butanol (HPLC; Macklin, Shanghai, China); Methanol (chromatographic grade; Aladdin, Shanghai, China); Ammonium acetate (LC-MS; Aladdin, Shanghai, China); Ammonia (LC-MS; Aladdin, Shanghai, China); Azithromycin in methanol (internal standard, 100 μg/mL, purity 98%, Ehrenstorfer, London, UK); Sodium carboxymethyl cellulose (Jiangsu Anxin Food Ingredients Mall, Nanjing, China); Sodium heparin (Leagene, Beijing, China); Endothelial Cell Medium (ECM, Scienncell, Carlsbad, CA, USA); Pancreatin (Gibco, Waltham, MA, USA); DPBS 1X (Gibco, Waltham, MA, USA); Matrigel matrix (Corning, New York, NY, USA).

### 3.2. Animals

Eighty-four Sprague Dawley (SD) rats of Specific Pathogen Free (SPF) grade were purchased from the Laboratory Animal Center of Southwest Medical University (Luzhou, Sichuan, China). The rats were kept in an animal room with a barrier system at temperature (20 ± 2 °C), humidity (50 ± 5%), and a 12 h light/dark cycle and were fasted for 18 h with free drinking water before administration. Experimental animal production license is No: SCXK (Chuan) 2018-17 and experimental animal use license number is No: SYXK (Chuan) 2018-065. All animal procedures were approved (No. SYXK202112–01) by the animal ethics committee of Luzhou New Drug Evaluation and Research Center (Luzhou, Sichuan, China).

The rats selected for the study weighed an average of 210 ± 23 g for males and 193 ± 14 g for females. They were randomly divided into seven groups, with each group consisting of twelve rats (six males and six females) sacrificed after administration periods of 0 (control group), 1, 3, 6, 12, 24, and 48 h. The rats were then given bitespiramycin by gavage (100 mg/kg). After 1, 3, 6, 12, 24 and 48 h post administration, rats were sacrificed by CO_2_ and the blood was collected from the inferior vena cava for further testing. Rat tissues, including lung, prostate, testis, bladder, ovary, uterus and brain were collected and frozen at −20 °C.

### 3.3. Patient Cerebrospinal Fluid Collection

Clinical trials were approved (No. KY20212078–C–1) by the Medical Ethics Committee of the Xijing Hospital of Fourth Military Medical University. Limited cerebrospinal fluid samples of six inpatients in the period of September 2021–January 2022 were collected at the 0 (control sample), 1, 5, 19 or 20 days after bitespiramycin treatment for further biochemical assay.

### 3.4. Standards and Sample Preparation for UHPLC–MS/MS

SPMs I, II, III and ISV-SPMs I, II, III standard stock solution (1 mg/mL) was prepared with acetonitrile-water (1:1, *v*:*v*) and stored at 4 °C, which was used for the calibration curves and the low, medium and high quality controls. ISV-SPMs II, III: 0.5 ng/mL (low), 9 ng/mL (medium) and 15 ng/mL (high), ISV-SPM I, SPMs I, II, III: 1 ng/mL (low), 18 ng/mL (medium) and 30 ng/mL (high) with acetonitrile-water (1:1, *v*:*v*). The concentrations of the low, medium, and high quality controls was determined based on the linear ranges of each component. The low quality controls (QCL) were set at approximately twice the lowest concentration within the linear range. The medium quality controls (QCM) were set at around 50% of the concentration within the linear range. The high quality controls (QCH) were set at about 70% of the concentration within the linear range.

The accurately weighed tissues were cut, fully dispersed, and homogenized using a glass grinder with the addition of pure water in different proportions: 1 mL/mg for brain, testis, and prostate; 2 mL/mg for uterus and ovary; 3 mL/mg for bladder; and 5 mL/mg for lung. The resulting tissue homogenate was then prepared along with plasma and cerebrospinal fluid for UHPLC-MS/MS analysis.

**Figure 5:**
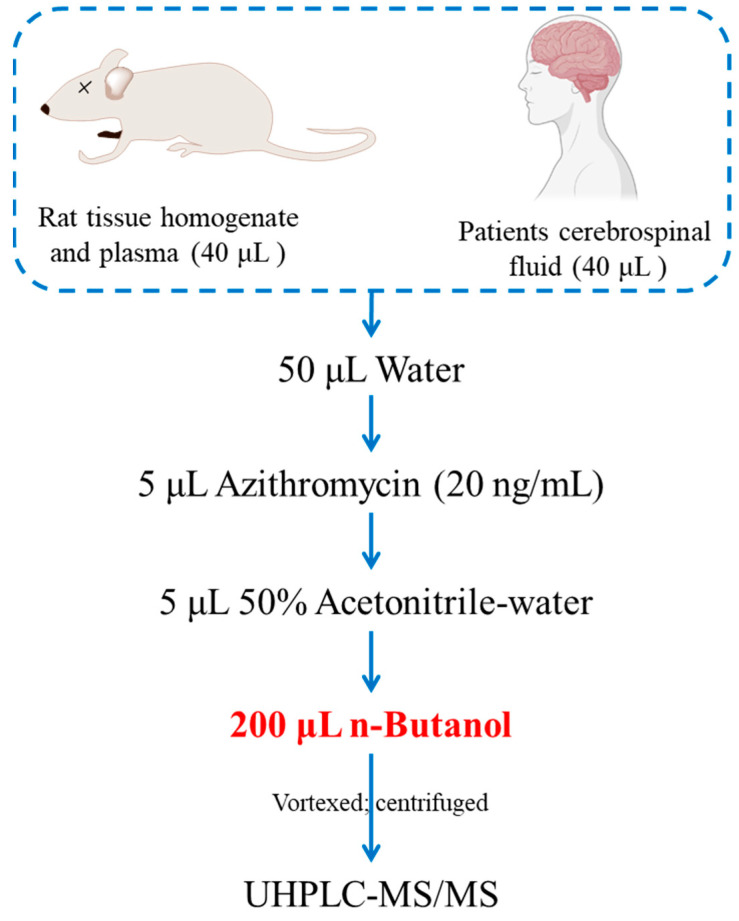
The sample preparation diagram.

40 μL of each biological sample (rat’s plasma or tissue homogenate, patients’ cerebrospinal fluid) was mixed with 50 μL of pure water, 5 μL of internal standard (Azithromycin, 20 ng/mL), 5 μL of 50% acetonitrile-water, and 200 μL of *n*-butanol. The mixture was vortexed for 1 min, sonicated for 2 min, and centrifuged at 13,000 rpm at 4 °C for 10 min. The supernatant was collected for UHPLC-MS/MS analysis (Figure 5).

### 3.5. UHPLC–MS/MS

UHPLC-MS/MS system was equipped with an UHPLC separation module (Acquity UPLC I-Class PLUS, Waters, Milford, MA, USA) and a triple Quad mass spectrometer. The Acquity BEH–C_18_ column (50 mm × 2.1 mm, 1.7 μm) combined with 5 mM ammonium acetate with 0.05% ammonia (pH 8.8) (A) and acetonitrile (B) was used for the separation with gradient elution (0–0.5 min, 5% B; 0.5–5 min, 5%–95% B; 5–9 min, 95% B). The flow rate was set at 0.3 mL/min, the injection volume was 2 μL, and the column temperature was set at 45 °C.

The mass spectrometry was performed on triple Quad 6500 plus mass spectrometer (Sciex, Boston, MA, USA). The parameters of the ES ion source in positive mode were set as follows: voltage 5500 V, curtain gas (CUR) 35 psi, spray gas (GS1) 50 psi, auxiliary heater (GS2) 50 psi, ionization temperature (TEM) 550 °C, the dwell-time values 0.2 s. The MS data recordings were carried out in multiple reaction monitoring (MRM) mode and the scanning time was 0.5 s. The declustering potential (DP), collision energy (CE) and transitions for each of the analytes and for internal standard were shown in Table 5.

### 3.6. Method Validation

Specificity and selectivity were validated by comparing blank samples, spiked samples, and samples after administration. Six standards spiked into blank bio-samples with gradient concentrations were determined. The concentrations of the analytes were subjected to linear regression with the ratio of the peak area of analytes to that of internal standard. LLOQ is defined as the lowest point of the linear regression curve with a precision less than 20%. Intra-day accuracy and precision were evaluated by continuously analyzing of six replicates of LLOQ, low, medium and high QC samples in a day. Inter-day accuracy and precision were assessed by analyzing of three batches of samples with the same material in three days. The precision should not be greater than 15% and the mean accuracy should be within ±15%, except for the LLOQ which can be within ±20% of the standard concentration.

Recovery and matrix effect were measured at low, medium, and high QC samples in six replicates. Matrix effect was assessed by comparing the concentrations of standards spiked bio-samples with that of solution spiked standards. Extraction recovery rate was evaluated by comparing the concentrations of pre-extraction samples with that of post-extraction samples.

The stability of bitespiramycin was investigated by comparing the concentrations of low, medium and high QC samples under the following four storage conditions: short-term stability of body fluid and tissues at 25 °C for 2 h (all the replicate samples were prepared within 2 h); long-term stability at –20 °C for 7 days; freeze-thaw stability for three cycles; autosampler stability at 4 °C for 24 h. The analytes were considered stable when the deviation was within ±15%.

## 4. Conclusions

An improved UHPLC–MS/MS method was developed and carefully validated, which simplified the sample preparation and improved the peak shape, resulting in significantly enhanced sensitivity and stability. This validated method was then successfully applied to the study of bitespiramycin distribution in rat plasma, tissues, and in human cerebrospinal fluid, in which bladder, prostate and cerebrospinal fluid of patients were examined for the first time. Moreover, bitespiramycin and its metabolites were detected for the very first time in rat brain, testis, bladder, and prostate, and in human cerebrospinal fluid.

Bitespiramycin was first proved to cross the blood-brain barrier, providing evidence of its therapeutic efficacy against intracranial infection. Distribution in lung and prostate with a higher concentration would directly contribute to its anti-pneumonia and anti-prostatitis efficacy. A promising strategy has been established for the treatment drug monitoring and elucidation of the underlying ADME of bitespiramycin, as well as other macrolide antibiotics.

## Figures and Tables

**Table 1:** Linear ranges of ISV-SPM I, II, III and SPM I, II, III in tissues and body fluid.

Tissues/Body Fluid	Linear Ranges	
	ISV-SPM I, SPM I, II, III	ISV-SPM II, III
Brain	1.2–72.8 ng/g	0.6–38.4 ng/g
Testis	0.6–800 ng/g	0.3–400 ng/g
Prostate	1.2–8000 ng/g	0.6–4000 ng/g
Uterus and ovary	1.8–12,000 ng/g	0.9–6000 ng/g
Bladder	2.4–16,000 ng/g	1.2–8000 ng/g
Lung	3.6–24,000 ng/g	1.8–12,000 ng/g
Plasma	0.6–4000 ng/mL	0.3–2000 ng/mL
Cerebrospinal fluid	0.6–38.4 ng/mL	0.3–19.2 ng/mL

**Table 2:** Comparison of LLOQs of SPMs and ISV-SPMs (ng/mL).

	ISV-SPM I	ISV-SPM I	ISV-SPM I	SPM I	SPM II	SPM III
LLOQ reported in [9]	4	12	18	4	12	18
LLOQ reported in [10]	1	1	1	1	1	1
LLOQ of this study	0.3	0.6	0.6	0.6	0.3	0.3

**Table 3:** The tissue-to-plasma concentration ratio (Ct/Cp) determined 24 h following oral administration of bitespiramycin at a dosage of 100 mg/kg.

	Brain	Lung	Prostate	Testis	Womb	Ovary	Bladder
ISV-SPM I	/	/	/	/	/	/	/
ISV-SPM II	0.31	83.10	78.81	2.70	30.66	26.28	12.35
ISV-SPM III	1.34	221.04	182.44	5.82	60.86	57.28	37.17
SPM I	0.22	89.29	53.50	5.60	62.18	35.75	31.27
SPM II	0.40	61.84	61.11	8.34	49.47	17.06	19.91
SPM III	0.26	69.01	34.46	1.95	29.59	14.19	9.87

**Table 4:** The concentrations of SPMs I, II, III and ISV-SPMs I, II, III in cerebrospinal fluid of patients.

No.	Sex	Age	Sampling Time *	Concentration (ng/mL)					
				ISV- SPM I	ISV- SPM II	ISV- SPM III	SPM I	SPM II	SPM III
1	Male	77	1	ND	0.47	0.60	ND	ND	ND
			5	ND	1.54	1.76	ND	ND	1.27
2	Male	44	1	ND	ND	ND	ND	ND	ND
			5	ND	0.31	0.42	ND	ND	0.75
3	Male	57	1	ND	ND	ND	ND	ND	ND
			5	ND	ND	ND	ND	0.99	0.67
4	Female	53	1	ND	ND	0.33	ND	1.36	0.83
			5	ND	1.73	1.83	ND	2.77	3.40
5	Male	43	1	ND	ND	ND	ND	0.74	ND
			5	ND	ND	ND	ND	1.13	0.86
6	Male	72	19	0.72	5.21	9.14	ND	4.69	13.00
			20	1.05	6.55	9.82	ND	5.61	11.90

* The days after continuous oral administration. ND: Not Detected.

**Table 5:** The declustering potential, collision energy and transitions for MS/MS detection.

Component	*m*/*z*	CE/V	DP/V
SPM I	843.7 → 174.2	43	80
SPM II	885.7 → 174.3	44	80
SPM III	899.7 → 174.4	46	80
ISV-SPM I	927.7 → 174.0	51	80
ISV-SPM II	969.8 → 174.1	48	80
ISV-SPM III	983.8 → 174.1	48	80
Azithromycin	749.7 → 591.6	42	90

## Data Availability

The data presented in this study are available in article and Supplementary Materials.

## Supplementary Materials

The following supporting information can be downloaded at: https://www.mdpi.com/article/10.3390/molecules29051037/s1.
