# Supplementary material for: Study of Bitespiramycin Distribution in Rats and Cerebrospinal Fluid of Patients by a Sensitive LC-MS/MS Method with Rapid Sample Preparation
Source: Molecules. 2024 Feb 28;29(5):1037. doi: 10.3390/molecules29051037 (PMC10934368; doi:10.3390/molecules29051037)
Supplement: Supplementary file 1 [file molecules-29-01037-s001.zip › molecules-2882502-supplementary.pdf]

Supporting Information of

Study of bitespiramycin distribution in rats and cerebrospinal fluid of patients by a sensitive LC-MS/MS method with rapid sample preparation

Yujie Zhang [1,†], Jingjie Cao [1,†], Jiahan Su [1], Tingting He [1], Qianru Wang [1], Feng Wei [2], Xin Guo [3], Qibing Mei [1,3,*] and Jing Zeng [1,*]

[1] School of Pharmacy, Southwest Medical University, Luzhou, China.
[2] Shanghai Tonglian Pharmaceutical Co. Ltd., Shanghai, China.
[3] Luzhou New Drug Safety Evaluation Research Center, Luzhou, China.
* Correspondence: authors: qbmei@swmu.edu.cn, zengjing@swmu.edu.cn
† These authors contributed equally to this work.

Table S1 The concentrations of SPMs I, II, III and ISV-SPMs I, II, III in rats' plasma after intragastric administration of 100 mg/kg of bitespiramycin (Mean ± SD, n = 6)

Components	Concentration（ng/mL）					
	1h	3h	6h	12h	24h	48h
ISV-SPM I	9.40±9.05	12.02±2.75	5.08±1.38	N.D.	N.D.	N.D.
ISV-SPM II	59.13±23.37	73.83±10.58	25.05±15.21	4.37±1.68	2.42±1.04	1.34±0.71
ISV-SPM III	29.45±10.34	37.42±4.12	13.15±8.48	2.24±1.23	1.34±0.72	0.71±0.53
SPM I	105.55±101.49	103.73±13.66	33.65±16.43	5.13±0.48	2.68±0.42	0.87±0.30
SPM II	728.33±540.15	658.00±128.69	338.67±53.09	101.42±13.34	48.38±10.33	14.78±5.06
SPM III	1728.33±1315.68	1766.67±635.25	970.00±264.78	363.50±78.90	126.80±28.97	47.12±17.00

Table S2 The concentrations of ISV-SPM I in rats' tissues after intragastric administration of 100 mg/kg of bitespiramycin (Mean ± SD, n = 6)

Tissues	Concentration（ng/g）					
	1h	3h	6h	12h	24h	48h
Brain	N. D.	0.75±0.34	N. D.	N. D.	N. D.	N. D.
Lung	64.50±40.24	217.1±45.20	129.20±48.23	37.74±7.07	28.60±11.65	7.36±1.31
Prostate	3.86±2.60	19.74±14.47	68.03±29.33	51.00±5.05	37.40±9.48	13.93±2.69
Testis	N. D.	2.00±0.65	1.68±0.56	0.82±0.33	0.90±0.36	N. D.
Womb	19.31±10.58	42.14±12.06	50.25±14.57	16.99±8.05	19.83±12.41	8.78±7.15
Ovary	23.92±16.64	71.55±25.72	127.05±48.24	29.27±6.65	21.01±7.53	9.16±3.70
Bladder	2.85±2.85	9.24±5.12	13.97±8.29	3.98±3.09	4.31±2.89	1.44±0.65

Table S3 The concentrations of ISV-SPM II in rats' tissues after intragastric administration of 100 mg/kg of bitespiramycin (Mean ± SD, n = 6)

Tissues	Concentration（ng/g）					
	1h	3h	6h	12h	24h	48h
Brain	2.10±0.89	4.69±2.04	3.00±1.12	0.35±0.21	0.75±0.85	N. D.
Lung	1407.00±278.56	2968.00±879.65	1727.00±332.09	218.60±69.47	201.10±77.54	48.59±5.26
Prostate	55.92±37.97	231.033±90.26	432.67±178.16	145.37±23.87	190.73±103.69	63.03±21.64
Testis	2.43±2.35	13.37±2.70	12.58±3.31	4.21±0.92	6.53±1.58	4.94±2.47
Womb	144.85±66.88	190.15±49.01	194.00±71.19	59.60±22.74	74.20±24.74	34.44±25.84
Ovary	188.45±99.16	265.10±76.33	382.75±87.20	56.95±12.89	63.60±30.06	24.79±10.73
Bladder	43.84±9.15	117.73±51.80	197.07±122.68	27.66±6.82	29.89±12.48	12.12±4.57

Table S4 The concentrations of ISV-SPM III in rats' tissues after intragastric administration of 100 mg/kg of bitespiramycin (Mean ± SD, n = 6)

Tissues	Concentration（ng/g）					
	1h	3h	6h	12h	24h	48h
Brain	2.56±1.08	5.24±2.14	4.40±2.31	1.05±0.58	1.79±1.89	0.54±0.16
Lung	1741.00±328.27	3573.00±1081.71	2444.00±660.74	318.70±109.78	296.20±134.05	73.77±8.99
Prostate	85.31±62.68	293.33±80.25	527.33±190.34	160.20±26.55	244.47±130.41	85.87±26.13
Testis	3.16±2.85	15.84±3.02	12.34±2.74	4.46±1.03	7.80±2.16	5.42±2.85
Womb	182.05±82.36	254.50±63.83	250.05±71.54	62.35±23.15	81.55±24.80	39.10±26.51
Ovary	299.00±170.41	358.40±92.98	472.50±108.94	60.95±12.37	76.75±37.06	31.60±13.07
Bladder	56.93±13.99	156.93±68.33	244.13±122.23	38.53±8.15	49.81±18.95	20.67±6.51

Table S5 The concentrations of SPM I in rats' tissues after intragastric administration of 100 mg/kg of bitespiramycin (Mean ± SD, n = 6)

Tissues	Concentration（ng/g）					
	1h	3h	6h	12h	24h	48h
Brain	N. D.	1.60±0.93	0.68±0.30	0.85±0.33	0.60±0.37	0.76±0.19
Lung	114.02±67.75	264.80±62.49	230.20±62.38	326.40±101.61	239.30±81.40	130.05±56.79
Prostate	47.34±46.94	147.53±59.90	251.97±136.15	318.67±70.04	143.37±29.00	73.07±8.61
Testis	9.73±9.91	43.13±22.42	17.15±4.15	21.15±5.68	15.02±3.17	11.55±2.16
Womb	288.25±162.62	624.00±133.77	795.50±195.03	183.95±56.90	166.65±79.97	88.40±52.58
Ovary	159.30±105.38	459.05±170.08	963.50±413.22	163.10±78.20	95.80±28.62	48.10±13.55
Bladder	81.13±48.35	139.40±70.63	175.00±108.28	105.18±80.16	83.80±38.39	53.11±15.34

Table S6 The concentrations of SPM II in rats' tissues after intragastric administration of 100 mg/kg of bitespiramycin (Mean ± SD, n = 6)

Tissues	Concentration（ng/g）					
	1h	3h	6h	12h	24h	48h
Brain	13.89±6.39	49.10±18.26	22.28±12.83	14.80±6.05	19.33±7.45	11.97±5.18
Lung	2598.00±1048.62	6728.00±2060.06	6662.00±1037.53	4260.00±1121.50	2992.00±695.75	1112.50±421.18
Prostate	882.63±712.74	3086.00±1207.64	5633.33±1966.06	4810.00±1264.83	2956.67±859.99	1450.33±175.52
Testis	100.70±88.77	581.67±207.52	729.00±186.97	482.67±68.44	403.33±63.39	241.13±41.26
Womb	2565.50±1064.35	3715.00±643.27	3929.00±909.20	2251.00±686.47	2393.50±972.49	1158.50±726.15
Ovary	1157.00±446.48	1786.00±491.13	2880.00±671.90	928.00±181.88	825.50±412.62	237.65±81.08
Bladder	945.73±481.23	1764.00±528.94	3448.67±1900.76	1302.00±514.03	963.33±489.50	569.67±315.48

Table S7 The concentrations of SPM III in rats' tissues after intragastric administration of 100 mg/kg of bitespiramycin (Mean ± SD, n = 6)

Tissues	Concentration（ng/g）					
	1h	3h	6h	12h	24h	48h
Brain	19.95±9.69	56.67±23.90	38.35±25.03	48.22±24.92	33.21±13.39	23.96±9.17
Lung	7846.00±2711.43	20250.00±2540.98	20150.00±2341.51	13300.00±3269.57	8750.00±1560.65	3700.33±1449.72
Prostate	996.07±766.55	3716.67±1554.32	6973.33±2019.69	6360.00±1464.19	4370.00±1004.69	2523.33±228.53
Testis	75.47±67.63	388.67±44.10	409.00±94.15	296.33±38.06	247.57±48.39	159.20±24.42
Womb	2645.00±1055.27	4940.00±1147.82	5410.00±1668.03	3346.50±956.57	3751.50±1422.66	1905.50±1243.33
Ovary	1736.50±624.10	3245.50±1007.69	5410.00±1113.43	2003.50±404.00	1799.50±912.43	580.95±194.30
Bladder	843.33±391.50	1818.67±502.55	4078.67±2147.62	1662.00±463.38	1252.00±415.61	656.00±146.73

Table S8 The concentration and penetrability of SPMs I, II, III and ISV-SPMs I, II, III in vitro (Mean ± SD, n = 6)

Time	Concentration（ng/ml）					
	ISV-SPM I	ISV-SPM II	ISV-SPM III	SPM I	SPM II	SPM III
2h	53.77±15.05	24.98±4.85	25.13±5.33	90.37±20.79	85.42±19.14	78.65±16.67
4h	90.37±15.81	40.38±5.50	40.43±6.46	128.33±23.64	124.15±21.30	113.38±18.93
8h	138.83±15.33	61.95±5.95	63.27±7.41	191.00±15.44	185.33±14.65	173.50±13.05
Time	Penetrability（%）					
	ISV-SPM I	ISV-SPM II	ISV-SPM III	SPM I	SPM II	SPM III
2h	8.07±2.26	7.49±1.45	7.54±1.60	13.56±3.12	12.81±2.87	11.80±2.50
4h	13.56±2.37	12.11±1.65	12.13±1.94	19.25±3.55	18.62±3.20	17.01±2.84
8h	20.82±2.30	18.59±1.79	18.98±2.22	28.65±2.32	27.80±2.20	26.03±1.96

Table S9. The tissue-to-plasma concentration ratio (Ct/Cp) determined 1 hours following oral administration of bitespiramycin at a dosage of 100 mg/kg.

	Brain	Lung	Prostate	Testis	Womb	Ovary	Bladder
ISV-SPM I	/	6.86	0.41	/	2.05	2.54	0.30
ISV-SPM II	0.04	23.80	0.95	0.04	2.45	3.19	0.74
ISV-SPM III	0.09	59.12	2.90	0.11	6.18	10.15	1.93
SPM I	/	1.08	0.45	0.09	2.73	1.51	0.77
SPM II	0.02	3.57	1.21	0.14	3.52	1.59	1.30
SPM III	0.01	4.54	0.58	0.04	1.53	1.00	0.49

Table S10. The tissue-to-plasma concentration ratio (Ct/Cp) determined 3 hours following oral administration of bitespiramycin at a dosage of 100 mg/kg.

	Brain	Lung	Prostate	Testis	Womb	Ovary	Bladder
ISV-SPM I	0.06	18.06	1.64	0.17	3.51	5.95	0.77
ISV-SPM II	0.06	40.20	3.13	0.18	2.58	3.59	1.59
ISV-SPM III	0.14	95.48	7.84	0.42	6.80	9.58	4.19
SPM I	0.02	2.55	1.42	0.42	6.02	4.43	1.34
SPM II	0.07	10.22	4.69	0.88	5.65	2.71	2.68
SPM III	0.03	11.46	2.10	0.22	2.80	1.84	1.03

Table S11. The tissue-to-plasma concentration ratio (Ct/Cp) determined 6 hours following oral administration of bitespiramycin at a dosage of 100 mg/kg.

	Brain	Lung	Prostate	Testis	Womb	Ovary	Bladder
ISV-SPM I	/	25.43	13.39	0.33	9.89	25.01	2.75
ISV-SPM II	0.12	68.94	17.27	0.50	7.74	15.28	7.87
ISV-SPM III	0.33	185.86	40.10	0.94	19.02	35.93	18.57
SPM I	0.02	6.84	7.49	0.51	23.64	28.63	5.20
SPM II	0.07	19.67	16.63	2.15	11.60	8.50	10.18
SPM III	0.04	20.77	7.19	0.42	5.58	5.58	4.20

Table S12. The tissue-to-plasma concentration ratio (Ct/Cp) determined 12 hours following oral administration of bitespiramycin at a dosage of 100 mg/kg.

	Brain	Lung	Prostate	Testis	Womb	Ovary	Bladder
ISV-SPM I	/	/	/	/	/	/	/
ISV-SPM II	0.08	50.02	33.27	0.96	13.64	13.03	6.33
ISV-SPM III	0.47	142.28	71.52	1.99	27.83	27.21	17.20
SPM I	0.17	63.63	62.12	4.12	35.86	31.79	20.50
SPM II	0.15	42.00	47.43	4.76	22.19	9.15	12.84
SPM III	0.13	36.59	17.50	0.82	9.21	5.51	4.57

Table S13. The tissue-to-plasma concentration ratio (Ct/Cp) determined 48 hours following oral administration of bitespiramycin at a dosage of 100 mg/kg.

	Brain	Lung	Prostate	Testis	Womb	Ovary	Bladder
ISV-SPM I	/	/	/	/	/	/	/
ISV-SPM II	/	36.26	47.04	3.69	25.70	18.50	9.04
ISV-SPM III	0.76	103.90	120.94	7.63	55.07	44.51	29.11
SPM I	0.87	149.48	83.99	13.28	101.61	55.29	61.05
SPM II	0.81	75.27	98.13	16.31	78.38	16.08	38.54
SPM III	0.51	78.53	53.55	3.38	40.44	12.33	13.92

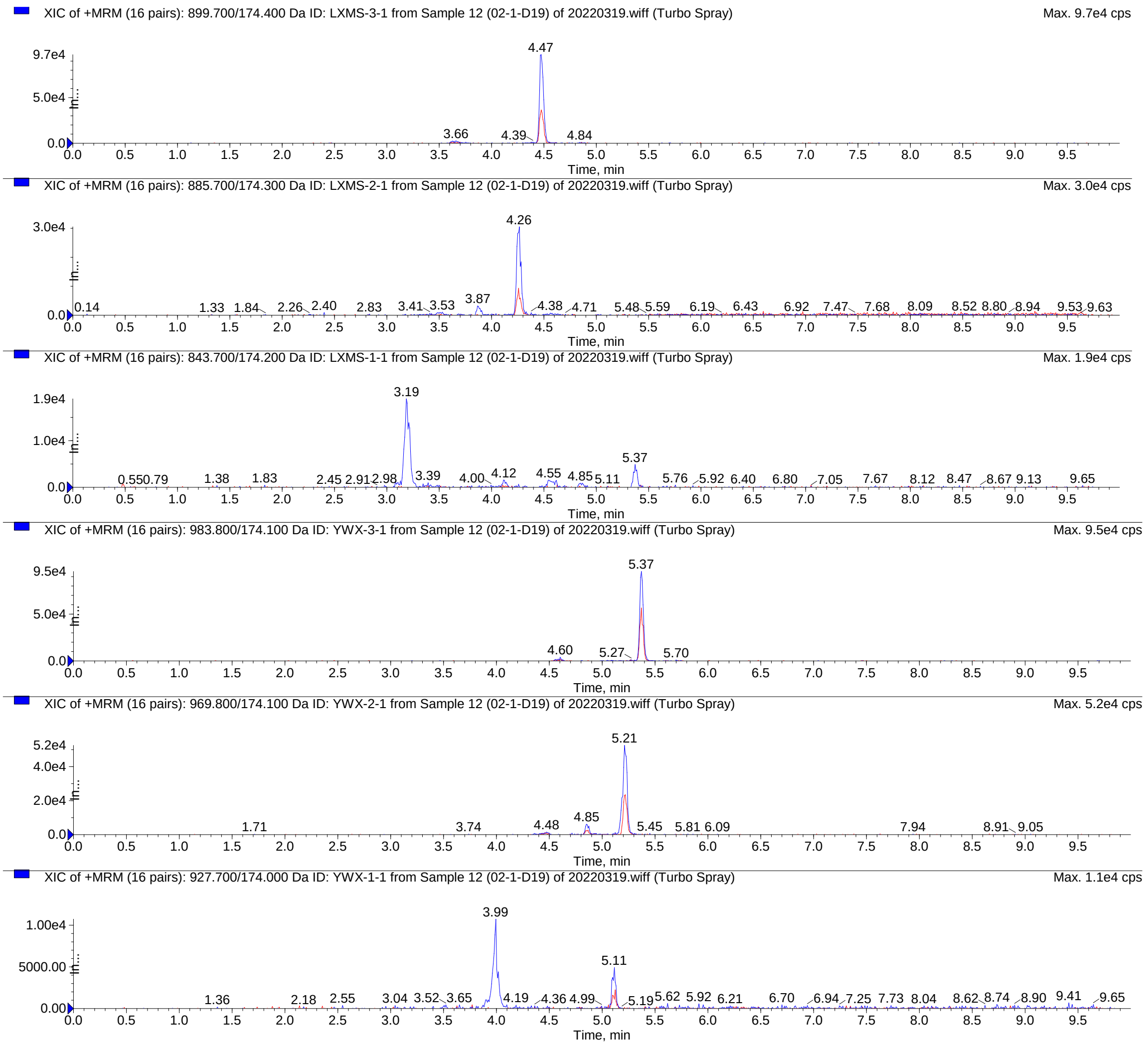

Figrue S1 The MRM chromatograms six components of the patient cerebrospinal fluid
